# Phytoextracts Used in the Treatment of Cryptosporidiosis and Giardiasis: Current State

**DOI:** 10.1007/s11686-026-01238-9

**Published:** 2026-03-12

**Authors:** Ardra Panavoor Namboodiri, Shahira Abdelaziz Ali Ahmed, Panagiotis Karanis

**Affiliations:** 1 Department of Biomedicine and Medical Diagnostics, Faculty of Health and Environmental Sciences, Auckland University of Technology, Auckland Central, Auckland, 1010 New Zealand; 2 Department of Parasitology, Faculty of Medicine, Suez Canal University, Ismailia, 41522 Egypt; 3 Faculty of Medicine, University of Cologne, University Hospital Cologne, Cologne, Germany; 4 Department of Basic and Clinical Sciences, University of Nicosia Medical School, 24005, CY-1700 Nicosia, Cyprus; 5 UNIC Athens, No. 17, 29th Street, Elliniko, 167 77 Athens, Greece

**Keywords:** *Cryptosporidium* species, *Giardia duodenalis*, Phytoextracts, Medicinal plants, In vitro, In vivo

## Abstract

Phytoextracts are safer, more affordable, and more readily available than conventional medications, making them promising anti-parasitic treatment options. *Cryptosporidium* species (spp.) and *Giardia duodenalis* are protozoan parasites responsible for cryptosporidiosis and giardiasis, respectively, primarily affecting immunocompromised individuals, children, and populations in developing countries. Although treatment options exist, many of them have significant side effects. The current review aims to evaluate the efficacy of phytoextracts tested against *Cryptosporidium* spp. and *G. duodenalis* infections. The current review indicated that the Asteraceae and Rutaceae families were the most extensively investigated sources of phytoextracts. Additionally, *Zingiber officinale* is the only extract to demonstrate efficacy against both parasites in in vivo studies. Furthermore, *Curcuma longa* and *Morinda royoc* showed the highest in vitro efficacy against *Cryptosporidium* spp. and *G. duodenalis*, respectively. These results underscore the potential of phytoextracts as alternative therapeutic modalities for the treatment of cryptosporidiosis and giardiasis.

## Introduction

*Cryptosporidium* is a genus of protozoan parasites that leads to the enteric disease cryptosporidiosis. This genus includes at least 47 species and more than 120 genotypes, of which 21 are known to cause human infections [1, 2]. The two common species that cause cryptosporidiosis are *Cryptosporidium parvum* and *Cryptosporidium hominis*, which account for around 90% of human infections [3]. Cryptosporidiosis is typically transmitted through the faecal-oral pathway [3]. It is one of the most prevalent food- and water-borne illnesses. *Cryptosporidium* infection is generally self-limiting in immunocompetent individuals [4]. This infection usually produces symptoms such as acute gastroenteritis, abdominal pain, and diarrhoea. However, in immunocompromised individuals, it commonly occurs as a chronic infection and can even be life-threatening [5].

Cryptosporidiosis imposes a substantially greater burden than is reflected in official surveillance data. Globally, the reported incidence is approximately three cases per 100,000 population; however, the true prevalence may be up to one hundred times higher, as many infections remain undetected or are misdiagnosed due to nonspecific clinical manifestations and limited diagnostic capacity [6] This underestimation is particularly pronounced in low-income settings, where inadequate water quality and poor sanitation enhance transmission [3, 7].

The World Health Organization estimates that tens of millions of infections occur annually, with the heaviest burden in sub-Saharan Africa and South Asia, where 10–20% of children under five years of age are affected [8]. Recent evidence shows that *Cryptosporidium* is widespread across Asia and remains a significant cause of diarrheal disease, particularly among children and immunocompromised individuals, with a prevalence of 8.1% over the past decade [9]. In the United States, around 7500 laboratory-confirmed cases are reported each year, although the actual number is thought to exceed 750,000 [10]. Similarly, European surveillance documents over 14,000 cases annually, largely concentrated in the United Kingdom, Germany, and Ireland [10]. Collectively, these marked regional disparities underscore cryptosporidiosis as a significant global public health and veterinary concern.

*Giardia* is another genus of protozoan parasites, with *Giardia duodenalis* being the species that causes giardiasis. This parasite has eight distinct genetic assemblages (A-H) [11]. However, only A and B are usually associated with human infections [11, 12]. This infection can be transmitted from person to person, zoonotically, through food or water [13]. *Giardia* infection, similar to cryptosporidiosis, is self-limited in immunocompetent patients [13, 14], resulting in diarrhoea and stomach pain [15], whereas in immunocompromised individuals it can become chronic, leading to malnutrition, malabsorption, and weight loss [15, 16]. The other vulnerable group is children under five years [17, 18], with a higher risk in developing countries [14].

Approximately 200 million people are infected with giardiasis each year, with an estimated 500,000 deaths attributed to the disease [19]. According to the World Health Organization, the burden is greatest in Asia, Africa, and Latin America, where around 200 million individuals develop symptoms, and nearly 500,000 new cases are reported annually [19]. In the European Economic Area (EU/EEA), 10,894 confirmed cases were recorded across 24 countries in 2022, corresponding to a notification rate of 3.9 per 100,000 population; Spain and Germany accounted for 46.6% of reported cases, while the highest notification rates were observed in Luxembourg, Belgium, and Spain [20].

Imunocompromised individuals who are at risk of cryptosporidiosis include cancer, HIV, organ transplant, and inflammatory bowel disease (IBD) patients. Immunosuppression may be inherent to the disease itself, as in cancer or HIV, or may result from the use of immunosuppressive medications, such as in organ transplant and IBD patients. *Cryptosporidium* is strongly associated with digestive cancer, specifically colorectal cancer [21, 22]. The risk is reported to be four times higher in patients with colon cancer in a study by Osman et al. (2017). Not only that, but an infection due to *Cryptosporidium* could potentially be carcinogenic in AIDS patients as well [21]. Cryptosporidiosis usually leads to chronic diarrhoea in HIV patients, which is fatal in children [23]. In organ transplant recipients, *Cryptosporidium* infection is a risk due to immunosuppression caused by immunosuppressant medication [24, 25]. In these patients, cryptosporidiosis could be asymptomatic, making it more dangerous [25]. Protozoal infections, specifically cryptosporidiosis, are also common in IBD patients due to the same issue of using immunosuppressant medications for treatment [26]. Furthermore, *Cryptosporidium* infection can also lead to a relapse in recovering patients [26]. Given the increased vulnerability of such individuals, new treatments for *Cryptosporidium* infections must be developed*.* Giardiasis places a disproportionate burden on immunocompromised individuals, who are more prone to persistent, severe, and clinically complex infections. In immunocompromised populations, giardiasis has been associated with treatment-refractory disease, chronic diarrhoea persisting beyond six months, increased clinical severity, and higher parasitic burdens, particularly among individuals with hypogammaglobulinemia, nephrotic syndrome, malignancies, and renal transplant recipients [27, 28]. Molecular epidemiological evidence indicates that patients undergoing corticosteroid therapy exhibit distinct *G. duodenalis* assemblage distributions and are approximately 36 times more likely than controls to harbor concurrent parasitic infections [16].

The current treatment for cryptosporidiosis includes the only FDA-licensed drug in the United States for this disease, nitazoxanide. However, nitazoxanide does not provide positive results to immunocompromised or malnourished individuals, such as patients with HIV. Nitazoxanide also causes many side effects, including nausea, vomiting, abdominal pain, diarrhoea, and anorexia [29–31]. Other drugs that can be used include paromomycin, clofazimine, miltefosine, or combination therapies. However, these drugs have their limitations as well. Paromomycin did not show a significant difference in results between the control and placebo groups, with side effects of nausea and abdominal pain [29, 31]. Additionally, nitazoxanide and paromomycin negatively affect the intestinal microbiota, further reducing its diversity [32]. Clofazimine was initially developed for treating leprosy; however, when tested for HIV-positive patients with *Cryptosporidium* infection, there were no significant effects on oocyst shedding, diarrhoea, or stool consistency [33–35]. Miltefosine is another antimicrobial used to treat parasitic infections, but it has side effects and limited efficacy [29, 31]. Combination therapy involved nitazoxanide or paromomycin with macrolides, rifamycin derivatives, or HAART (Highly Active Antiretroviral Therapy). However, clinical trials showed that HAART did not significantly reduce the *Cryptosporidium* infection rate among patients receiving HAART compared with those not receiving HAART [36]. Therefore, extensive controlled trials must validate their efficiency in treating immunocompromised individuals. High cure rates were also seen in spiramycin and garlicin, another combination therapy. The study, however, did not include HIV patients, and the immune function of patients was also overlooked [31, 37]. There are currently no vaccines or chemotherapeutic drugs against *Cryptosporidium* infections [30, 31].

In giardiasis treatment, six drugs are used as first-line therapy: 5-nitroimidazoles, benzimidazoles, quinacrine, furazolidone, paromomycin, and nitazoxanide [12]. Metronidazole, the most common, is effective but has neurotoxic and genotoxic effects, along with nausea, metallic taste, abdominal pain, and diarrhoea [12, 38]. Tinidazole, another nitroimidazole, has high cure rates with milder side effects [12, 29]. Albendazole and mebendazole, both benzimidazoles, are alternatives. Albendazole is as effective as metronidazole but has fewer side effects [12]. While generally safe, hepatic toxicity has been reported after a single dose of albendazole [40]. Drug resistance to albendazole and mebendazole is a growing concern [11]. Nitazoxanide, a newer treatment, has inconsistent cure rates, reducing its reliability [39]. There are no vaccines available yet for *Giardia*, and increasing drug resistance is leading to more treatment failures [41].

*Giardia* resistance to the main antiparasitic agents (metronidazole, tinidazole, nitazoxanide) is increasingly reported, but large-scale prevalence estimates remain scarce. In Stockholm (2008–2020), 102 of 4285 cases (2.4%) were nitroimidazole-refractory giardiasis, with a markedly higher and increasing proportion among infections acquired in India (64/545; 12%) [42]. A global narrative review has reported that as many as 50% of giardiasis cases become resistant to standard 5-nitroimidazole regimens, with the highest failure rates observed among travellers returning from endemic areas [43]. Similar concerns are described in European and Latin American case series, where metronidazole failure rates have been reported to exceed 10% in some cohorts, although precise national-level figures are lacking [44].

Considering the previous limitations of chemical drugs for market anti-cryptosporidiosis and anti-giardiasis, scientists turned to traditional medicine. Traditional medicine refers to the use of plants and plant extracts (phytoextracts) to treat diseases. Phytoextracts are extracts derived from plants containing active compounds. Plants have been used for the treatment of diseases for a long time and are still being used in Africa and Asia. They have recently been tested against parasite infections, and many have provided promising results. Using plants’ phytoextracts as remedies has many advantages, (i) less toxic; (ii) abundantly available; (iii) These compounds are environmentally friendly and have broad therapeutic potential [45]. Several bioactive components, such as flavonoids, glycosides, saponins, tannins, alkaloids, terpenes, and steroids, create the pharmacological properties of phytoextracts [46]. Phytoextracts from many plants have shown antiparasitic properties. For instance, *Glycyrrhiza glabra* L. (liquorice) and *Syzygium aromaticum* (cloves) have shown activity against *Plasmodium*,* Babesia*,* Leishmania*, and *Theileria* parasites, while *Matricaria chamomilla* L. (chamomile) have also exhibited antiparasitic properties against parasites such as *Leishmania amazonensis* and *Leishmania infantum* [47, 48]. Thus, phytoextracts hold potential as alternative treatments for *Cryptosporidium* spp. and *Giardia duodenalis* infections.

Given the increasing rates of drug resistance in *Cryptosporidium* and *Giardia* infections and the limited efficacy of existing medications, a comprehensive review of the phytoextracts employed is both timely and essential. This review critically synthesises and evaluates the current state of research on plant-derived phytoextracts used to treat cryptosporidiosis and giardiasis, highlighting their antiparasitic efficacy, phytochemical profiles, mechanisms of action, safety considerations, and analytical techniques.

## Methodology

### Search Strategy

The PubMed database was searched with no restrictions for language or year of publication on the 11^th^ of August 2024. To evaluate the potential of plant-based remedies (phytoextracts) for treating *Cryptosporidium* spp. and *Giardia duodenalis* infections. The literature search strategy was limited primarily to title/abstract/keyword. The title/abstracts from the retrieved articles were reviewed to determine if they met the inclusion criteria, and the articles were then selected accordingly.

Two separate searches were conducted: one for publications about *Cryptosporidium* species and phytoextracts and the other for publications about *Giardia duodenalis* and phytoextracts. The search was performed separately to ensure that no relevant articles were missed.

For *Cryptosporidium* spp., the search was performed using the following MeSH terms/key words: (“*Cryptosporidium* “[Mesh] OR “Cryptosporidiosis“[Mesh] OR “*Cryptosporidium parvum*“[Mesh] OR *Cryptosporidium* [tw] OR *Cryptosporidiosis*[tw] OR “*Cryptosporidium* Infection*“[tw] OR “*Cryptosporidium parvum*“[tw] OR “*Cryptosporidium hominis*“[tw]) AND (“Phytochemicals“[Mesh] OR “Herbal Medicine“[Mesh] OR “Plants, Medicinal“[Mesh] OR “Plant Extracts“[Mesh] OR “Phytotherapy“[Mesh] OR Phytoextract*[tw] OR Phytochemical*[tw] OR “Plant Extract*“[tw] OR “Plant-Derived Compound*“[tw] OR “Plant Bioactive Compound*“[tw] OR Phytonutrient*[tw] OR “Herbal Medicin*“[tw] OR “Medicinal Plant*“[tw] OR “Medicinal Herb*“[tw] OR “Pharmaceutical Plant*“[tw] OR Phytotherapy[tw] OR “Herbal Therapy“[tw]).

The search for *Giardia duodenalis* was done using the following MeSH terms/key words: (“*Giardia*“[Mesh] OR “Giardiasis“[Mesh] OR “*Giardia lamblia*“[Mesh] OR *Giardia*[tw] OR *Giardiasis*[tw] OR “*Giardia* Infection*“[tw] OR “*Giardia lamblia*“[tw] OR “*Giardia duodenalis*“[tw]) AND (“Phytochemicals“[Mesh] OR “Herbal Medicine“[Mesh] OR “Plants, Medicinal“[Mesh] OR “Plant Extracts“[Mesh] OR “Phytotherapy“[Mesh] OR Phytoextract*[tw] OR Phytochemical*[tw] OR “Plant Extract*“[tw] OR “Plant-Derived Compound*“[tw] OR “Plant Bioactive Compound*“[tw] OR Phytonutrient*[tw] OR “Herbal Medicin*“[tw] OR “Medicinal Plant*“[tw] OR “Medicinal Herb*“[tw] OR “Pharmaceutical Plant*“[tw] OR Phytotherapy[tw] OR “Herbal Therapy“[tw]).

### Eligibility Criteria

Articles with titles containing the words *Cryptosporidium* spp. or *Giardia duodenalis* in association with phytoextracts were selected and screened as part of the eligibility for inclusion in the literature review. Secondary screening for full PDF text was performed on the primary screened articles.

Following a full-text review, pertinent data were retrieved to identify the phytoextracts and their interactions with *Cryptosporidium* spp. and *Giardia duodenalis*. The extracted data included the following details: authors name, year of publication, study location, and plant-specific information (i.e., plant species, plant family, the extracted plant part, and the solvent media for the extract), each study characteristics (i.e., whether the research was in vitro or in vivo, the use of fractionation, and the specific fractions tested), the anti-parasitic activity of phytoextract (IC_50_ and EC_50_ values, parasite morphological/numerical changes, reductions in (oo) cysts shedding, change in parasite load, clinical improvements such as reduced diarrhoea), and phytoextract toxicity (LD_50_ values and documented side effects).

Publications were excluded if they lacked an abstract, were not entirely textual, were written in non-English languages, were case series, reviews, or contained confusing data (such as inconsistent data, poorly cited sources, or identical conclusions as another paper by the same author).

### Definitions

- Phytoextract: A concentrated substance obtained from plants through various extraction methods that were derived from plant tissues like leaves, roots, flowers, or seeds [49].
- Assay: A laboratory procedure to measure the activity or concentration of a drug substance [50].
- Bioactive components: Chemical compounds in plant extracts that influence biological systems or diseases [51].
- Combination therapy: Using multiple drugs simultaneously to increase treatment efficacy [52].
- Dissolved media: Solvents or liquids used to extract plant compounds for testing [53].
- Plant families: Groups of related plants classified together based on shared structural, genetic, and reproductive characteristics [54].
- Endemic species: Species native to and found only in a specific geographic location [55].
- Flora: Plant life native to a particular region or habitat [56].
- Fractions (in chemical extraction): Separated components of a plant extract with specific chemical properties [57].
- EC_50_: The concentration of an extract or compound that produces 50% of its maximum effect in each test system [58].
- IC_50_: The concentration of the extract that inhibits 50% of a specific biological or biochemical “in vitro studies” [59].
- LD_50_: A lethal dose to 50% of a large number of test animals of a particular species, “in vivo studies” [60]. LD_50_ is typically expressed in mg of substance per kg of body weight (mg/kg).

### Data Analysis

**Fig. 1:**
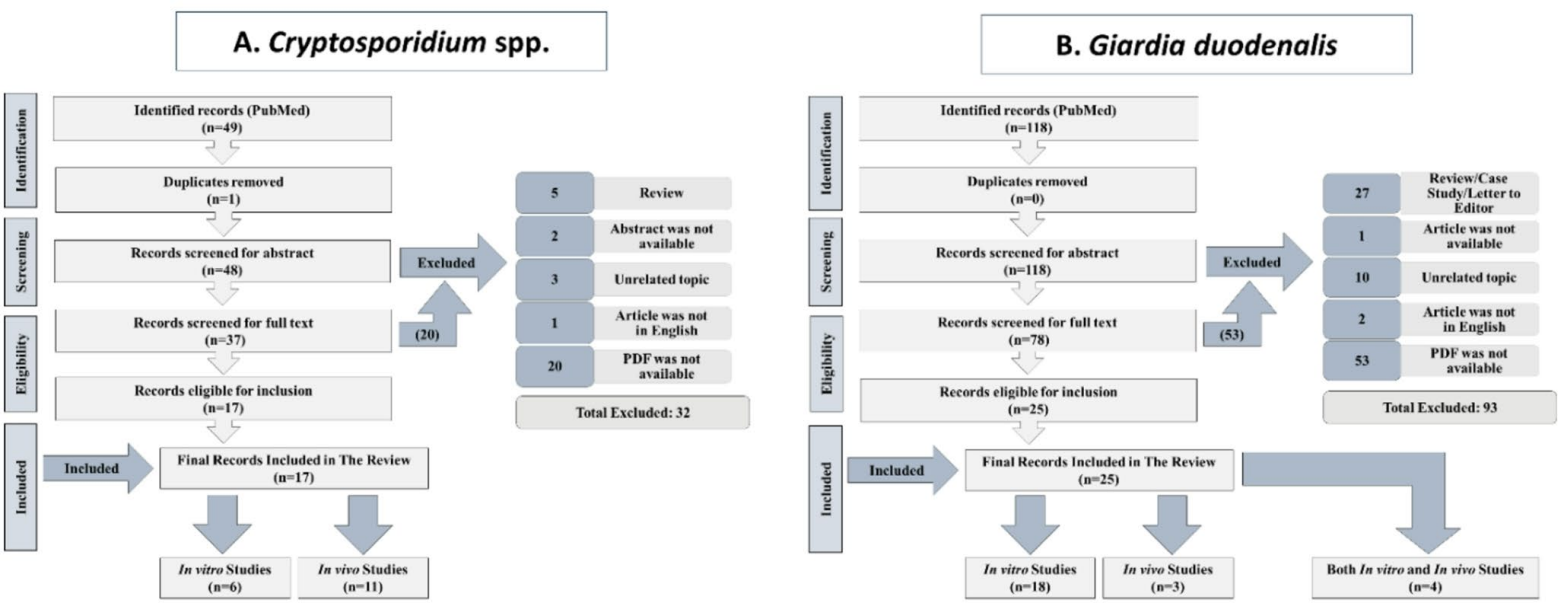
Flow chart of literature search and studies selection

The data is represented separately for *Cryptosporidium* spp. and *Giardia duodenalis*, with detailed descriptions in the respective sections. The present review contained one figure and four tables. Figure 1 depicts the process of retrieved articles identification, screening, and selection for *Cryptosporidium* spp. and *Giardia duodenalis*. Table 1 summarises the phytoextracts that were investigated in vitro against *Cryptosporidium* spp. Table 2 summarises the phytoextracts utilised in vitro against *Giardia duodenalis*. Table 3 details the in vivo experiments of phytoextracts interactions with *Cryptosporidium* spp., and Table 4 summarises the in vivo studies of phytoextract interactions with *Giardia duodenalis*.

**Table 1:** In vitro toxicity of phytoextracts to *Cryptosporidium parvum*

St. No.*	Plant species	Family	Country	Plant parts	Dissolved media	Fractions	IC_50_ Value (µg/mL)	Phytoextract activity	References
1	*Allium sativum* L.	Amaryllidaceae^F1^	India^C1^	Bulb/Cloves	Methanol 70%	N/A	N/A	N/A	[67]
2	*Artemesia judaica*	Amaryllidaceae^F1^	Egypt^C2^	Aerial part	Ethanol 70%	Phenolic	123.83	Inactive	[78]
						Terpenoid	233.24	Inactive	
						Flavonoids	47.27	Moderate	
1	*Boswellia serrata Roxb.*	Burseraceae^F3^	India^C1^	Stem	Ethanol 70%	N/A	N/A	N/A	[67]
	*Centella asiatica* L. *Urban (Gotukola)*	Apiaceae^F4^		Leaf			N/A	N/A	
3	*Cichorium intybus cv. Spadona*	Asteraceae^F2^	Denmark^C3^	Leaf	2% Formic acid in a mixture of Methanol (4/1; v/v)	Sesquiterpene lactones	139.7	Inactive	[79]
				Root			660.3	Inactive	
1	*Cucurbita pepo* L.	Cucurbitaceae^F5^	India^C1^	Seed	Water	N/A	N/A	N/A	[67]
	*Curcuma longa* L.	Zingiberaceae^F6^		Rhizome			0.003†	Active	
	*Embelia ribes* Burm. F.	Primulaceae^F7^		Fruit	Methanol 70%		0.0781†	Active	
	*Glycyrrhizaglabra* L.	Primulaceae^F7^		Fruit			N/A	N/A	
4	*Lotus pedunculatus*	Fabaceae^F8^	Norway^C4^	Leaf	1% DMSO	Condensed tannins	16.63–60.04**	Active	[71]
1	*Moringa oleifera* Lam.	Moringaceae^F9^	India^C1^	Leaf	Water	N/A	N/A	N/A	[67]
	*Nigella sativa* L.	Ranunculaceae^F10^		Seed			0.1413†	Active	
5	*Olea europaea* L.	Oleaceae^F11^	Italy^C5^	Fruit	Ethanol 70%	N/A	361	Inactive	[80]
4	*Onobrychis viciifolia*	Fabaceae^F8^	Norway^C4^	Herbage	1% DMSO	Condensed tannins	16.63–60.04**	Active	[71]
6	*Pinus sylvestris*	Pinaceae^F12^	Norway^C4^	Bark	Acetone 70%	N/A	244.6	Inactive	[81]
					Methanol 80%		279.1	Inactive	
1	*Piper nigrum* L.	Piperaceae^F13^	India^C1^	Fruit	Acetone/Hexane	N/A	0.0781†	Active	[67]
4	*Ribes nigrum*	Grossulariaceae^F14^	Norway^C4^	Leaf	1% DMSO	Condensed tannins	16.63–60.04**	Active	[71]
1	*Thymus vulgaris* L.	Lamiaceae^F15^	India^C1^	Seed	Water	N/A	N/A	N/A	[67]
	*Tribulus terrestris* L.	Zygophyllaceae^F16^		Entire Plant			N/A	N/A	
4	*Trifolium repens*	Fabaceae^F8^	Norway^C4^	Flower	1% DMSO	Condensed tannins	16.63–60.04**	Active	[71]
1	*Vitex negundo* L.	Lamiaceae^F15^	India^C1^	Leaf	Water	N/A	N/A	N/A	[67]
4	*Vitis vinifera*	Vitaceae^F17^	Norway^C4^	Seed	1% DMSO	Condensed tannins	16.63–60.04**	Active	[71]
6	22	17	5	12	7	5		9 Active	

St. No.: Study number; N/A: Not Available; DMSO: Dimethyl sulfoxide; *The phytoextracts were sorted in alphabetical order, therefore some studies were duplicated and mentioned using their starting numbers in the table; ** High toxicity did not allow for exact calculations (in the form of HCT-8 cell death); †: Calculated and converted to µg/mL; ^F1, F2, F3,^etc.: Families of phytoextracts were acronymic and superscripted by alphabetical-numerical categorization to reflect the number of phytoextracts families used; ^C1, C2, C3,^ etc.: Location of collection of phytoextracts were acronymic and superscripted by alphabetical-numerical categorization to reflect the number of countries

**Table 2:** In vitro toxicity of phytoextracts to *Giardia duodenalis*

St. No.	Plant Species	Family	Country	Plant Parts	Dissolved media	Fractions	IC_50_ Value (µg/mL)	Phytoextract activity	References
1	*Aegle marmelos*	Rutaceae^F1^	India^C1^	Fruit (unripe)	Water	N/A	N/A	N/A	[87]
2	*Ageratum conyzoides*	Asteraceae^F2^	Thailand^C2^	LP	Ethanol	N/A	463.08	Inactive	[88]
				LW-P			45.67	Moderate	
				FW			166	Inactive	
				FP			96	Moderate	
				FW-P			207	Inactive	
				EO-LW–P			35	Moderate	
				EO- FP			89.33	Moderate	
				LW			130	Inactive	
3	*Ageratum conyzoides*	Asteraceae^F2^	Austria^C3^	Leaves	DMSO	Methyllacarol	N/A	N/A	[89]
	*Aglaia odorata*	Meliaceae^F3^		Stembark		Aglafoline			
4	*Allium sativum*	Amaryllidaceae^F4^	UK^C4^	Bulb	Sterial media	Whole Garlic	300†	Inactive	[68]
						Allyl alcohol	7†	Active	
						Allyl mercaptan	37†	Moderate	
						Methyl propyl sulphide	250†	Inactive	
						Allyl methyl sulphide	550†	Inactive	
						Diallyl sulphide	1300†	Inactive	
						Methyl sulphide	1300†	Inactive	
						Diallyl disulphide	100†	Moderate	
						Dimethyl disulphide	200†	Inactive	
						Methyl propyl disulphide	300†	Inactive	
						Dipropyl disulphide	450†	Inactive	
5	*Allium sativum (diallyl trisulfide)*	Amaryllidaceae^F4^	Mexico^C5^	Bulb	Water	N/A	686	Inactive	[69]
6	*Allium sativum* L.	Amaryllidaceae^F4^	China^C6^	Bulb	Culture medium	N/A	14	Moderate	[70]
7	*Alocasia indica*	Araceae^F5^	India^C1^	Leaves	Water	N/A	4.12	Active	[90]
					95% Ethanol		4.65	Active	
8	*Annona cherimola*	Annonaceae^F6^	Mexico^C5^	Leaves	10% Ethanol	Ethanol Extract	137.3	Inactive	[91]
						DCM	131.7	Inactive	
						EtOAc	37.2	Moderate	
						Aqueous Residual	134.7	Inactive	
9	*Bursera fagaroides* var. *fagaroides*	Burseraceae^F7^	Mexico^C5^	Bark	Hexane	Burseranin	16.14†	Moderate	[92]
						5′-desmethoxy-β-peltatin-A-methylether	1.80†	Active	
						Acetylpodophyllotoxin	0.97†	Active	
						Podophyllotoxin	1.61†	Active	
10	*Elaeodendron trichotomum*	Celastraceae^F8^	Mexico^C5^	Leaves, Stembark, Root	CH2Cl2 and Methanol	N/A	0.44	Active	[93]
11	*Fragaria ananassa* variety Elsanta	Rosaceae^F9^	UK^C4^	Fruit	0.1% Formic Acid	N/A	N/A	N/A	[72]
3	*Glycosmis angustifolia*	Rutaceae^F1^	Austria^C3^	Leaves	DMSO	Methyldambullin	N/A	N/A	[89]
	*Glycosmis chlorosperma*			Leaves		Sakambullin			
	*Glycosmis pentaphylla*			Leaves		Arborine			
	*Glycosmis sapindoides*			Rootbark		Kokusagenine			
	*Glycosmis sapindoides*			Leaves		Iso-gamma-fagatine			
	*Glycosmis sapindoides*			Leaves		Arborine			
	*Glycosmis trichanthera*			Stembark		5-Hydroxynoracronycine			
	*Glycosmis trichanthera*			Leaves		Methylgerambullin			
	*Glycosmis trichanthera*			Rootbark		Dictamnine			
	*Glycosmis trichanthera*			Stembark		Yukocitrine			
12	*Helianthemum glomeratum*	Cistaceae^F10^	Mexico^C5^	Aerial Parts	10% Methanol-Water	Methanol Extract	95.6	Moderate	[94]
						8 (quercetin)	26.4	Moderate	
						1 (tiliroside)	17.4	Moderate	
						2 (kaempferol-3-O-(3″,6″-	81.5	Moderate	
						di-O-E-p-coumaroyl)-β-D-glucopyranoside)			
						3 (astragalin)	47.5	Moderate	
						4 (quercitrin)	24.2	Moderate	
						5 (isoquercitrin)	47.5	Moderate	
						6 (kaempferol)	8.7	Active	
						7 (coumaric acid)	85.4	Moderate	
13	*Helianthemum glomeratum*	Cistaceae^F10^	Mexico^C5^	Aerial Parts	100% Methanol	Po1 Fraction	N/A	N/A	[95]
						Po2 Fraction			
						Po3 Fraction			
						Po4 Fraction			
						Po5 Fraction			
11	*Hippophae rhamnoides*	Elaeagnaceae^F11^	Finland^C7^	Fruit	0.1% Formic Acid	N/A	N/A	N/A	[72]
14	*Larrea tridentata*	Zygophyllaceae^F12^	USA^C8^ and Mexico^C5^	Leaves	100% Methanol	1 (Nordihydroguairetic acid)	N/A	N/A	[96]
						2 (3’-O-methylnordihydroguairetic acid)			
						3 (Nor-3’-demethoxyisoguaiacin )			
						4 (Analogues of compound 3 with tetrahydronaphthalene ring)			
						5 (Analogues of compound 3 with tetrahydronaphthalene ring)			
						6 (Analogues of compound 3 with tetrahydronaphthalene ring)			
						7 (3-hydroxy-4-epi-larreatricin)			
						8 ((7R 7’R)-7 7’-bis(4’ 3, 4-trihydroxyphenyl)-(8R, 4-trihydroxyphenyl)-(8R, 8’S)-8 8’-dimethyl tetrahydrofuran))			
						9 (flavanol)			
3	*Micromelum* cf. *minutum*	Rutaceae^F1^	Austria^C3^	Leaves	DMSO	Microminutine	N/A	N/A	[89]
15	*Morinda royoc*	Rubiaceae^F13^	Mexico^C5^	Roots	50% Methanol	Methanol-water residual	1.13	Active	[97]
						Hexane	0.08	Active	
						Dichloromethane	1.1	Active	
						Ethyl acetate	1.09	Active	
16	*Persea americana*	Lauraceae^F14^	Mexico^C5^	Seed	Chloroform and 100% Ethanol	CHCl₃	0.634	Active	[98]
						EtOH	0.486	Active	
17	*Phaseolus vulgaris*	Fabaceae^F15^	Mexico^C5^	Seed	Acetic acid	N/A	209	Inactive	[99]
					80% Methanol		275	Inactive	
					Acidified water		390	Inactive	
18	*Piqueria trinervia*	Asteraceae^F2^	Mexico^C5^	Aerial Parts	0.4% DMSO	Red Oil	5.66	Active	[100]
						Fraction 1	5.03	Active	
						Fraction 2	3.38	Active	
19	*Punica granatum*	Lythraceae^F16^	Mexico^C5^	Peel	30% Ethanol	Polyphenolic Fraction	179	Inactive	[101]
11	*Punica granatum* L.	Lythraceae^F16^	UK^C4^	Fruit	0.1% Formic Acid	N/A	N/A	N/A	[72]
20	*Rhamnus cathartica*	Rhamnaceae^F17^	Iran^C9^	Bark	100% Methanol	N/A	74.46	Moderate	[102] **
11	*Ribes nigrum* L. variety 8982-6	Grossulariaceae^F18^	UK^C4^	Fruit	0.1% Formic Acid	N/A	N/A	N/A	[72]
	*Rubus chamaemorus* L.	Rosaceae^F9^	Finland^C7^						
	*Rubus fruticosus*	Rosaceae^F9^	UK^C4^						
21	*Rubus liebmannii*	Rosaceae^F9^	Mexico^C5^	Aerial Parts	Ethanol	Ethanol Extract	11.75	Moderate	[103]
11	*Rubus stellatus× R. arcticus*	Rosaceae^F9^	Finland^C7^	Fruit	0.1% Formic Acid	N/A	N/A	N/A	[72]
22	*Sambucus ebulus*	Adoxaceae^F19^	Iran^C9^	Fruit	100% Methanol	N/A	47,940†	Inactive	[104] **
11	*Sorbus aucuparia* L. c.v. Sahharnaja	Rosaceae^F9^	Finland^C7^	Fruit	0.1% Formic Acid	N/A	N/A	N/A	[72]
	*Vaccinium myrtillus* L. variety Berkeley	Ericaceae^F20^	UK^C4^						
	*Vaccinium vitis-idaea*	Ericaceae^F20^	Finland^C7^						
3	*Zanthoxylum simulans*	Rutaceae^F1^	Austria^C3^	Rootbark	DMSO	Zanthobungeanine	N/A	N/A	[89]
22	34	20	9	11	13	64		17 Active	

St. No.: Study number; N/A: Not Available; LW: Leaves of White Flowered Plants; LP: Leaves of Purple Flowered Plants; LW-P: Leaves of White-Purple Flowered Plants; EO: Essential oils; FW: White Flowers; FP: Purple Flowers; FW-P: Flowers from White-Purple Flowered Plants; UK: United Kingdom; USA: United States of America; DMSO: Dimethyl sulfoxide; *The phytoextracts were sorted in alphabetical order, therefore some studies were duplicated and re-mentioned using their starting numbers in the table; †: Calculated and converted to µg/mL; ^F1, F2, F3,^ etc.: Families of phytoextracts were acronymic and superscripted by alphabetical-numerical categorization to reflect the number of phytoextracts families used; ^C1, C2, C3,^ etc.: Location of collection of phytoextracts were acronymic and superscripted by alphabetical-numerical categorization to reflect the number of countries. ** These studies used cysts for phytoextracts investigation

**Table 3:** In vivo experiments of phytoextracts with *Cryptosporidium* spp.

*St. No.	Plant Species	Family	Country	Plant Parts	Animal tested	Number of Animals Used	Extraction Method	Adm. route	Fractions	LD_50_ (mg/kg) /EC_50_ (µg/mL)	Efficacy**	Toxicity / Symptoms	Reference
1	*Allium sativum*	Amaryllidaceae^F1^	Romania^C1^	Aerial part	Swine	240	Ground and mixed with cereal flour	Oral	N/A	N/A	Not Eff.	No	[109]
2	*Allium sativum*	Amaryllidaceae^F1^	Egypt^C2^	Bulb	Swiss Albino Mice	82	Distilled water	IG	N/A	N/A	Eff. ^1^	No / Mild degenerative lesions in the gastric lining and lymphoid hyperplasia in the spleen	[73]
3	*Arabidopsis thaliana*	Brassicaceae^F2^	Indonesia^C3^	Leaves	*M. coucha* Mice	57	Water/Methanol 100%	Oral	N/A	N/A	Not Eff.	No	[110]
1	*Artemisia absinthium*	Asteraceae^F3^	Romania^C1^	Bulb	Swine	240	Ground and mixed with cereal flour	Oral	N/A	N/A	Not Eff.	No	[109]
4	*Calendula officinalis*	Asteraceae^F3^	Romania^C1^	Aerial part	Swine	240	Ground and mixed with cereal flour	Oral	N/A	N/A	Not Eff.	No	[111]
5	*Castanea sativa*	Fagaceae^F4^	Italy^C4^	Bark	Calves	20	20% propylene glycol solution and Milk	Oral	Tannin	N/A	Eff. ^2^	No	[112]
6	*Cocos nucifera* L.	Arecaceae^F5^	Egypt^C2^	Fruit	Albino Mice	60	Water, n-hexene, Ethanol 70%	Oral	N/A	N/A	Eff.^3, 4, 5^	No	[113]
3	*Diospyros sumatrana*	Ebenaceae^F6^	Indonesia^C3^	Leaves	*M. coucha* Mice	57	Water/Methanol 100%	Oral	N/A	N/A	Eff. ^3^	No	[110]
7	*Ficus carica*	Moraceae^F7^	Egypt^C2^	Leaves	Swiss Mice	49	Methanol 70%	Oral	N/A	N/A	Eff. ^3, 6^	No	[114]
8	Nekka-Rich (*Castanopsis cuspidate* and *Quercus acuta*)	Fagaceae^F4^	Japan^C5^	Bark	Calves	6	Milk	Oral	N/A	N/A	Eff. ^2, 3^	No	[115]
7	*Olea europaea*	Oleaceae^F8^	Egypt^C2^	Leaves	Swiss Mice	49	Methanol 70%	Oral	N/A	N/A	Eff.^3, 4^	No	[114]
3	*Piper betle*	Piperaceae^F9^	Indonesia^C3^	Leaves	*M. coucha* Mice	57	Water/Methanol 100%	Oral	N/A	N/A	Not Eff.	No	[110]
9	*Podocarpus elongatus* (Aiton) L’ Hér. ex-Pers	Podocarpaceae^F10^	Egypt^C2^	Leaves	Swiss Albino Mice	56	Methanol 25 mg%	Oral	N/A	LD_50_: >4000	Eff.^3^	No	[116]
	*Podocarpus gracilior* (Pilg.)												
	*Podocarpus macrophyllus* (Thunb.)												
2	*Punica granatum*	Lythraceae^F11^	Egypt^C2^	Peels	Swiss Albino Mice	82	Water and Methanol 100%	IG	N/A	N/A	Eff. ^1^	No/ Mild degenerative lesions in the gastric lining and lymphoid hyperplasia in the spleen	[73]
10	*Punica granatum*	Lythraceae^F11^	Israel^C6^	Peels	Calves	41	Milk	Oral	N/A	N/A	Eff. ^2, 3^	No	[117]
4	*Satureja hortensis*	Lamiaceae^F12^	Romania^C1^	Aerial part	Swine	240	Ground and mixed with cereal flour	Oral	N/A	N/A	Not Eff.	No	[111]
5	*Schinopsis spp.*	Anacardiaceae^F13^	Italy^C4^	Wood	Calves	20	20% Propylene glycol solution and Milk	Oral	Tannin	N/A	Eff. ^2^	No	[112]
11	*Thymus vulgaris*	Lamiaceae^F12^	Turkey^C7^	Leaves	Sprague Dawley Rats	66	Glycerine and Water, 1:3 ratio	Oral	N/A	N/A	Eff. ^3^	No	[118]
2	*Zingiber officinale*	Zingiberaceae^F14^	Egypt^C2^	Rhizomes	Swiss Albino Mice	82	Methanol 100%	IG	N/A	N/A	Eff. ^1^	No/ Mild degenerative lesions in the gastric lining and lymphoid hyperplasia in the spleen	[73]
11	19	14	7	8	4	-	9	2	1		12 Eff.	19 No Toxicity	

St. No.: Study number; N/A: Not Available; Adm. route: Administration route; IG: Intra-gastric; *The phytoextracts were sorted in alphabetical order, therefore some studies were duplicated and mentioned using their starting numbers in the table; ^F1, F2, F3,^ etc.: Families of phytoextracts were acronymic and superscripted by alphabetical-numerical categorization to reflect the number of phytoextracts families used; ^C1, C2, C3,^ etc.: Location of collection of phytoextracts were acronymic and superscripted by alphabetical-numerical categorization to reflect the number of countries; **Efficacy was classified as either effective (Eff.) or not effective (Not Eff.) based on the findings of each study; consequently, the efficacy type was expressed using superscripted numerals as follows: ^1^ Improved spleen health, ^2^ Reduced diarrhoea, ^3^ Reduction in oocyst shedding, ^4^ Improved intestinal architecture, ^5^ Fewer Caspase-3 Positive Cells (an indicator of tissue damage), ^6^ No significant toxicity and low LD_50_

**Table 4:** In vivo experiments of phytoextracts with *Giardia duodenalis*

*St. No.	Plant Species	Family	Country	Plant Parts	Animal tested	Number of Animals Used	Extraction Method	Adm. route	Fractions	LD_50_ (mg/kg) /EC_50_ (µg/mL)	Efficacy	Toxicity/Side effects	Reference
1	*Alocasia indica*	Araceae^F1^	India^C1^	Leaves	Rats	40	Water	Oral	N/A	LD_50_: >2000	Eff. ^1^	No / Mild GIT discomfort and reduction in GIT motility	[90]
							Ethanol						
2	*Anethum graveolens*	Apiaceae^F2^	Iraq^C2^	Leaves	Humans (paediatric)	28	Water	Oral	N/A	N/A	Eff. ^2^	No	[119]
3	*Cinnamon zeylanicum*	Lauraceae^F3^	Egypt^C3^	Rhizomes	Albino Rats	30	Distilled water	Oral	N/A	N/A	Eff. ^2^	No / Mild mucosal irritation and reduced goblet cell numbers.	[74]
4	*Phaseolus vulgaris*	Fabaceae^F4^	Mexico^C4^	Seed	Wistar Rats	6	Acetic acid	IG	N/A	LD_50_: 250	Not Eff.	No / One death due to broncho-aspiration	[99]
							80% Methanol						
							Acidified Water						
5	*Rhamnus cathartica*	Rhamnaceae^F5^	Iran^C5^	Bark	Balb/c Mice	30	Methanol	IG	N/A	EC_50_: 12	Eff. ^2^, ^3^	No	[102]
6	*Rubus liebmannii*	Rosaceae^F6^	Mexico^C4^	Aerial Parts	Balb/c Mice and Sprague-Dawley rats	30	Ethanol	Oral	Ethanol Extract	LD_50_:>5,000†	Eff. ^3^	No	[103]
7	*Yucca baccata*	Asparagaceae^F7^	Mexico^C4^	Stem	Gerbils	42	Water and n-butanol	Oral	N/A	N/A	Eff. ^2^	Yes / 3 deaths & overall weight loss	[120]
3*	*Zingiber officinale*	Zingiberaceae^F8^	Egypt^C3^	Rhizomes	Albino Rats	30	Distilled water	Oral	N/A	N/A	Eff. ^2^	No / Mild GIT discomfort and reduced goblet cell numbers	[74]
7	8	8	5	6	4	-	7	2	1		7 Eff.	7 No Toxicity	

St. No.: Study number; N/A: Not Available; Adm. route: Administration route; IG: Intra-gastric; GIT: Gastrointestinal Tract; *The phytoextracts were sorted in alphabetical order, therefore some studies were duplicated and mentioned using their starting numbers in the table; ^F1, F2, F3,^ etc.: Families of phytoextracts were acronymic and superscripted by alphabetical-numerical categorization to reflect the number of phytoextracts families used; ^C1, C2, C3,^ etc.: Location of collection of phytoextracts were acronymic and superscripted by alphabetical-numerical categorization to reflect the number of countries; **Efficacy was classified as either (Eff.) or not effective (Not Eff.) based on the findings of each study; consequently, the efficacy type was expressed using superscripted numerals as follows: ^1^ Reduced diarrhoea, ^2^ Reduction in trophozoite counts, ^3^ No significant toxicity and low LD_50_

The included studies used different data representations. A single expression that unified several terms was used to unify the range of terminologies of the included studies of the present review. The scientific names of the phytoextracts were included in their respective tables in alphabetical order. Using alphabetical-numerical categorisation, the phytoextracts’ comparable families were superscripted with acronyms (e.g., F1, F2, F3, etc.). The location of phytoextracts collection was acronymic and superscripted by alphabetical-numerical categorisation to reflect the number of countries (e.g., C1, C2, C3, etc.).

Depending on the type of biological assay, the test system, and the specific context in which the phytoextracts are assessed, the precise thresholds for classifying a phytoextract’s anti-parasitic efficacy varied across the included studies. Therefore, for in vitro studies, the IC_50_ and EC_50_ criteria were followed, categorising the phytoextracts’ activities into “active, moderate, and inactive” groups in the current investigation.

A lower IC_50_ indicates higher extract potency (activity), while a higher IC_50_ indicates less activity. The reference guidelines [61–64] have categorised the IC_50_ of a phytoextract into:

- Active: IC_50_ ≤ 10 µg/mL.
- Moderate: IC_50_ between 10 and 100 µg/mL.
- Inactive: IC_50_ > 100 µg/mL.

The lower the EC_50_, the more potent the extract. The EC_50_ is classified according to [64–66]:

- Active: EC_50_ ≤ 10 µM.
- Moderate: EC_50_ between 10 and 100 µM.
- Inactive: EC_50_ > 100 µM.

Since few studies have used LD_50_ and EC_50_ for in vivo studies, the efficacy of phytoextracts was classified as either “effective” or “not effective” based on the results of each study. The effectiveness of the phytoextracts for *Cryptosporidium* spp. was categorised based on the study’s objectives: (i) improved spleen health, (ii) reduced diarrhoea, (iii) reduced oocyst shedding, (iv) improved intestinal architecture, (v) fewer Caspase-3 Positive Cells (an indicator of tissue damage), (vi) no significant toxicity and low LD_50_. Phytoextracts’ efficacy against *Giardia duodenalis* was assessed based on the study’s objectives and described as (i) reduced diarrhoea, (ii) lower trophozoite counts, (iii) no notable toxicity, and a low LD_50_.

In certain studies, the IC_50_, EC_50_, and LD_50_ values were not specified; therefore, they were listed as unavailable (N/A) in the tables.

## Results

### Search Results and Study Selections

A total of 167 articles were collected and presented, of which one article was removed as duplicate. Of the 166 titles and abstracts screened, 115 were retained for eligibility evaluation at the full text screening stage. The studies included in the current review were published between 1962 and 2024, spanning a 60-year period.

Overall, 42 original studies were included in the review, with 17 articles on *Cryptosporidium* spp. (Fig. 1a) and 25 on *Giardia duodenalis* (Fig. 1b). The selection process and flowchart of the literature search are shown in (Fig. 1a and b).

### Phytoextracts and Parasite Data Stratification

*Cryptosporidium* spp. have been investigated for the toxicity of phytoextracts in 17 studies. Of these, 6 (35.3%) were performed in vitro, while 11 (64.7%) were utilised in vivo assays. In the case of *Giardia duodenalis*, 25 studies explored the toxicity of various phytoextracts, with 18 (72%) conducted in vitro, and 3 (17%) utilised in vivo assays, including four studies that incorporated both in vitro and in vivo experiments (Tables 1, 2 and 3, and 4).

There have been 78 plant species and 39 families reported, with Rutaceae (8 species) and Asteraceae (5 species) accounting for the majority. *Allium* was the most common plant genus recorded, with *Allium sativum* being the most common species and represented in six investigations (Tables 1, 2 and 3, and 4).

For the preparation of extracts, various plant parts were utilised, including leaves, aerial parts, bark, seeds, peel, roots, rhizomes, fruit, stems, shoots, bulbs, herbage, and the entire plant. The solvents that were used in different studies as dissolving media included water (both distilled and acidified), alcohols (ethanol at concentrations of 30%, 70%, methanol at 10%, 50%, 70%, 80%, and 100%), dimethyl sulfoxide (DMSO), formic acid (at concentrations of 0.1%, 0.4%, and 2%), and acetone (70%) (Tables 1, 2 and 3 and 4).

Researchers in the included studies employed different assays to examine the phytoextract’s effectiveness against *Cryptosporidium* spp. and *Giardia duodenalis in vitro*, in vivo, or in combination. The methodology was separated based on the study’s objectives, which included the phytoextract of interest, the parasite, and their interactions in vivo and in vitro, whereas the names of the assays used by investigators in the methodology, the assay aim, and each underlying technique were shown in detail.

The plant species investigated for *Cryptosporidium* spp. were different from those investigated for *Giardia duodenalis*. The difference became more pronounced between the in vitro and the in vivo experiments. Similar species were used for both parasites, in which the in vitro phytoextracts were *Allium sativum* [67–70] and *Ribes nigrum* [71, 72], and the in vivo phytoextracts were *Zingiber officinale* [73, 74]. It was impossible to evaluate if *Allium sativum* and *Ribes nigrum* were effective against *Cryptosporidium* spp. and *Giardia duodenalis*, respectively, because the authors did not publish the IC_50_ values for both in vitro studies. However, *Zingiber officinale* demonstrated efficacy against both parasites in vivo.

The flora in the current review included 13 countries, focusing on phytoextracts investigated for toxicity against *Cryptosporidium* spp., both in vitro and in vivo. The countries with the most studies reported were India (12), Norway (5), and Egypt (4). Several endemic plant species from India have been reported, including *Curcuma longa* L., *Piper nigrum* L., *Embelia ribes*,* Nigella sativa* L., and others (Tables 1 and 3). The most investigated plant families against *Cryptosporidium* spp. were Asteraceae (4), Lamiaceae (3), and Amaryllidaceae (3). Plants in the Asteraceae family included *Artemisia judaica*, *Artemisia absinthium*, and *Calendula officinalis*. Additionally, the plant most studied for its cryptosporicidal properties is *Allium sativum*, which grows in Egypt and India.

Regarding *Giardia duodenalis*, the flora in the current analysis included 15 countries that examined the toxicity of phytoextracts in vitro and in vivo. The countries with the most reported phytoextracts were Mexico (7), the United Kingdom (UK) (5), and Finland (4). Mexico had the highest representation of endemic flora, with species like *Annona cherimola*,* Bursera fagaroides*,* Persea americana*, and *Rubus liebmannii.* The most frequently cited plant families were Rutaceae (8) > Rosaceae (6) > and Asteraceae (3). The Rutaceae family contained plants such as *Glycosmis trichanthera*, *Glycosmis sapindoides*, *Micromelum cf. minutum*, and *Zanthoxylum simulans*. Similar to the studies on *Cryptosporidium* spp., *Allium sativum* was the most investigated phytoextract for its giardicidal activity, having been studied in Mexico, the United Kingdom, and China.

Our findings indicate that, although research endeavours have been undertaken in developed nations, developing countries are focused on developing new treatment options for cryptosporidiosis and giardiasis using plant-derived natural resources. Although both diseases have been researched in nearly the same number of nations, studies on giardiasis were more common.

With over 20,000 species spread throughout almost every kind of habitat on Earth, the Asteraceae family is the biggest and most varied group of flowering plants [75]. With over 160 genera and 2100 species, the Rutaceae is a large family with a diverse variety of morphological features and a nearly worldwide distribution [76, 77]. Their abundance can explain the widespread usage of the Asteraceae and Rutaceae families in the included studies.

### Phytoextracts and in Vitro Studies by Parasite

#### In Vitro Toxicity of Phytoextracts to *Cryptosporidium* spp.

About six studies investigated the effects of different phytoextracts on *Cryptosporidium* spp. in vitro. The in vitro assays investigated 22 different phytoextracts belonging to 13 different families for their cryptosporicidal activity. The only *Cryptosporidium* spp. that were investigated in the included studies was *C. parvum*, with no studies on other species.

Five countries-Egypt, Norway, India, Denmark, and Italy-studied the interaction of various phytoextracts with *Cryptosporidium* spp. India contributed the most significant proportion of the phytoextracts introduced (Table 1).

The growth inhibition assay was the most used method for analysing phytoextract and *Cryptosporidium* interactions in vitro in the included studies, accounting for 66.6% of investigations [67, 71, 79, 81]. Wet mount assays with ‘6-diamidino-2-phenylindol/propidium iodide (DAPI/PI) staining (50%) [71, 79, 81] were used to assess cell viability before the addition of phytoextracts. Furthermore, 66.6% of included research used Water-Soluble Tetrazolium 1 (WST-1) cell viability assays [71, 79–81] and 50% used dose-response curves [67, 78, 80] to evaluate cytotoxicity in response to phytoextract. *Cryptosporidium* was primarily detected using polymerase chain reaction (PCR) (66.6%) [67, 71, 78, 81]. Furthermore, high-performance liquid chromatography-mass spectrometry (HPLC-MS) was used in 66.6% of studies to identify phytochemicals present in the phytoextracts [67, 79–81].

The IC_50_ values of the cryptosporicidal phytoextracts ranged from 0.0032 µg/mL (rhizome of *Curcuma longa* L.) [67] to 660.3 µg/mL (root extract of *Cichorium intybus cv. spadona*) [79] (Table 1). The phytoextracts that were potent against *C. parvum* according to the reported IC_50_ were *Curcuma longa* L. (0.0032 µg/mL), *Piper nigrum* L. (0.0781 µg/mL), *Embelia ribes* Burm. f. (0.0781 µg/mL) et *Nigella sativa* L. (0.1413 µg/mL) [67]. The flavonoid fraction of *Artemisia judaica* (47.27 µg/mL) was a phytoextract that displayed moderate anti-*Cryptosporidium* activity [78].

Phytoextracts that were completely ineffective against *Cryptosporidium* spp. were the phenolic and terpenoid fractions of *Artemisia judaica* (123.83 and 233.24 µg/mL, respectively), *Cichorium intybus* (139.7 µg/mL), *Olea europaea* (361 µg/mL), and *Pinus sylvestris* (244.6 µg/mL) [78–81]. *Lotus pedunculatus*,* Ribes nigrum*,* Onobrychis viciifolia*,* Trifolium repens*, and *Vitis vinifera* were not studied enough to give exact IC_50_ values; they were assumed to be between 16.63 and 60.04 µg/mL [71] (Table 1).

The fractionation of phytoextracts into constituents, such as flavonoids, phenolics, terpenoids, sesquiterpenes, lactones, and condensed tannins, was only performed in three investigations. According to the observed IC_50_, the flavonoids of *Artemisia judaica* were the fraction exhibiting the most excellent efficacy [78] (Table 1).

The effective anti-*Cryptosporidium* activity of *Curcuma longa* L., *Piper nigrum* L., *Embelia ribes* Burm. f., and *Nigella sativa* L. could be attributed to their abilities to induce oxidative stress, disrupt parasite metabolism, and trigger apoptosis. This is caused by the bioactive compounds: curcumin, piperine, embelin, and thymoquinone, respectively, which exhibit antioxidant, anti-inflammatory, and cytotoxic properties (67). Curcumin is a polyphenol, piperine is an alkaloid, and embelin and thymoquinone are quinones. Phenolic compounds, alkaloids, and quinones have all been proven to display anti-parasitic activity [82–84].

Phytochemical investigations of *Curcuma longa* L. have identified the presence of alkaloids, terpenoids, flavonoids, steroids, carbohydrates, and saponins [85]. These bioactive compounds exert antiparasitic effects through diverse mechanisms: alkaloids compromise parasite membranes and inhibit critical enzymes, thereby impairing growth and survival; terpenoids disrupt membrane integrity and uncouple mitochondrial oxidative phosphorylation; flavonoids interfere with mitochondrial energy metabolism, reducing oxidative phosphorylation and parasite viability; and steroids promote the generation of reactive nitrogen species [86]. Collectively, these mechanisms likely underpin the observed antiparasitic activity of *Curcuma longa* L.

#### In Vitro Toxicity of Phytoextracts to *G. duodenalis*

A total of 25 studies investigating the effects of various phytoextracts on *Giardia duodenalis* were identified. A total of 34 plant species and 20 families, predominantly Rutaceae (8 species), have been documented. *Allium* and *Glycosmis* were the most common plant genera investigated for giardicidal impact, being the most common species and represented in three investigations (Table 2).

Nine countries-Austria, the United Kingdom, Finland, Iran, India, Thailand, China, the United States of America, and Mexico-studied the interaction of various phytoextracts with *Giardia duodenalis*. Mexico contributed the most significant proportion of the phytoextracts introduced (Table 2).

 In vitro phytoextract and *Giardia duodenalis* interaction tests were conducted using the Trypan blue exclusion assay in 31.8% of the included studies [68, 69, 72, 87, 92, 97, 99], while 3-(4,5-dimethylthiazol-2-yl)-2,5-diphenyltetrazolium bromide (MTT) assays were also performed in 31.8% of studies [69, 91, 92, 95, 98, 100, 102]. Post-treatment morphology was examined using scanning electron microscopy (SEM) [68, 69, 92, 101] and transmission electron microscopy (TEM) [68, 69, 88], which were used in 18.1% and 13.6% of the experiments, respectively. Furthermore, 22.7% of research used nuclear magnetic resonance (NMR) spectroscopy [89, 92, 93, 95, 96] and 31.8% used HPLC-MS [89, 91, 93, 96, 97, 101, 102] for phytochemical analysis of extracts, while 9% used molecular docking techniques to anticipate interactions between bioactive chemicals and *Giardia* target proteins [72, 91].

Apart from two research studies that utilised cysts exclusively [104] and another that combined cysts and trophozoites [102] for treatment investigation, all in vitro investigations examined the phytoextracts on *Giardia* trophozoites. The IC_50_ values were primarily utilised in these studies to investigate phytoextracts against *Giardia*. In a few investigations, the EC_50_ was employed [89, 96].

The IC_50_ values of giardicidal phytoextracts ranged from 0.08 µg/mL (hexane fraction) of *Morinda royoc* [97] to 47,940 µg/mL of *Sambucus ebulus* [104] (Table 2). The phytoextracts that were potent and had a significant giardicidal impact were the allyl alcohol fraction of *Allium sativum* (IC_50_: 7 µg/mL) [68], the water and ethanol extracts of *Alocasia indica* (IC_50_: 4.12 and 4.65 µg/mL respectively) [90], *Elaeodendron trichotomum* (IC_50_: 0.44 µg/mL) [93], the kaempferol fraction of *Helianthemum glomeratum* (IC_50_: 8.6 µg/mL) [94], all fractions of *Morinda royoc* (IC_50_: 0.08–1.13 µg/mL) [97], both fractions of *Persea americana* (IC_50_: 0.63 and 0.48 µg/mL) ( [98], all fractions of *Piqueria trinervia* (IC_50_: 3.38–5.66 µg/mL) [100], and *Bursera fagaroides* var. *fagaroides* (IC_50_: 0.97–1.80 µg/mL) [92].

Other phytoextracts that have been reported with moderate giardicidal activity were *Ageratum conyzoides* (35–96 µg/mL) [88], fractions of *Allium sativum* (14–100 µg/mL respectively) [68, 90], EtOAc fraction of *Annona cherimola* (37.2 µg/mL) [91], burseranin fraction of *Bursera fagaroides* var. *fagaroides* (16.14 µg/mL) [92], all other fractions of *Helianthemum glomeratum* (17.4–95.6 µg/mL) [94], *Rhamnus cathartica* (74.46 µg/mL) [102], and *Rubus liebmannii* (11.75 µg/mL) [103] (Table 2).

Phytoextracts that were completely ineffective against *Giardia duodenalis* were *Allium sativum (diallyl trisulfide)* (686 µg/mL) [69], *Phaseolus vulgaris* (209–390 µg/mL) [99], *Punica granatum* (179 µg/mL) [101], and *Sambucus ebulus* (47,940 µg/mL) [104] (Table 2).

In two studies, the EC_50_ values were used and have ranged from 7.71 µM (aglafoline fraction of *Aglaia odorata)* to 188 µM (analogues of nor-3’-demethoxyisoguaiacin fraction of *Larrea tridentata*) [89, 96].

All the effective phytoextracts contain poly-phenolic compounds, such as flavonoids, lignin, tannins [73, 90, 92, 94, 98, 100, 105, 106]. This is likely the reason for their anti-*Giardia* activity, since phenolic compounds have been proven to have anti-parasitic properties [82]. Phytochemical studies have shown that *Morinda royoc* roots are rich in anthraquinone-type compounds, with morindone and soranjidiol identified as major constituents of the extract and hexane fraction. These anthraquinones are strongly associated with antigiardial activity, as demonstrated by the potent inhibitory effect of the hexane fraction against *Giardia lamblia* trophozoites in vitro (IC_50_ = 0.08 µg/mL), without compromising the viability of human cell lines [97]. Anthraquinone compounds are known to exert antiparasitic and antimicrobial effects through mechanisms linked to oxidative stress induction: upon activation they can generate reactive oxygen species (ROS) such as singlet oxygen, superoxide anions, and hydroxyl radicals, which cause oxidative damage to cellular components and disrupt parasite physiology [107, 108]. This oxidative assault can lead to depolarization of mitochondrial membranes and trigger programmed cell death pathways, ultimately compromising parasite survival. Taken together, the presence of morindone and soranjidiol in *M. royoc* likely contributes to its traditional use and observed antiprotozoal properties, warranting further investigation into their specific modes of action and therapeutic potential.

### Phytoextracts and in Vivo Studies by Parasite

#### In Vivo Experiments of Phytoextracts with *Cryptosporidium* spp.

In the in vivo assays, 19 different plants belonging to 14 families were studied, with *Allium sativum* and *Punica granatum* tested in two separate studies. The animals used in the in vivo experiments varied among mice, swine, calves, and rats. The effectiveness of the phytoextracts investigated was measured based on the findings from the tested animals in each study. Phytoextract treatment efficacy was determined based on a reduction in *Cryptosporidium*-caused diarrhoea, a reduction in oocyst shedding, improvement in treated intestinal architecture, improved spleen health, a decrease in caspase-3 positive cells, which are indicators of tissue damage, or the absence of significant toxicity with low LD_50_ values measured.

In research on *Cryptosporidium* spp. in vivo, serum chemistry analyses were conducted in 27.2% of investigations to assess biological effects and safety [112, 114, 118]. Treatment efficacy was determined using faecal egg count reduction tests (FECRT) (63.3%) [109, 111–113, 116–118]. In 45.5% of investigations included, histopathology examinations (H & E) were done to assess tissue status post-treatment and compare it to the control [73, 110, 113, 114, 116].

Among the phytoextracts studied, *Podocarpus* spp., *Olea europaea* (with *Ficus carica*), Nekka-Rich, and *Thymus vulgaris* have been reported to improve the investigated animals significantly [114–116, 118]. Notably, *Podocarpus* spp. exhibited an LD_50_ greater than 4000 mg/kg [116]. Plants such as *Allium sativum*,* Castanea sativa*,* Cocos nucifera* L., *Diospyros sumatrana*,* Ficus carica*,* Schinopsis* spp., and *Zingiber officinale* also showed effectiveness, while others produced no effectiveness (Table 3). Moreover, *Punica granatum* has been proven effective in two studies, which is why it was only counted as one phytoextract when calculating the total number of effective phytoextracts [73, 117].

The effective doses varied widely depending on the plant species and preparation methods, ranging from 10 g/day for Nekka-Rich in calves to 100 mg/kg/day for *Podocarpus* spp. in mice [115, 116]. Overall, *Thymus vulgaris* and *Punica granatum* demonstrated effectiveness at lower concentrations [117, 118].

In-vivo *Cryptosporidium* studies typically used acute and subacute toxicity assays for safety, with haematological and serum chemistry analysis to examine biological effects on target animals. Treatment efficacy was assessed using MIC tests, cytotoxicity assays, and FECRT. Histological investigation (H & E staining) and Ziehl-Neelsen staining were employed to evaluate the effects on tissue and identify the oocysts of *Cryptosporidium*, respectively.

All the phytoextracts studied were stated to be non-toxic (Table 3). However, some phytoextracts have been reported to induce side effects in different animals, such as *Allium sativum*, *Punica granatum*, and *Zingiber officinale* [73]. The side effects of the phytoextracts were the formation of mild degenerative lesions in the gastric lining and lymphoid hyperplasia in the spleen [73].

#### In Vivo Experiments of Phytoextracts with *Giardia duodenalis*

In the in vivo assays, eight plants related to 8 different families—*Rubus liebmannii*, *Rhamnus cathartica*,* Zingiber officinale*,* Cinnamon zeylanicum*,* Alocasia indica*, * Phaselus vulgaris*,* Yucca baccata*, and *Anethum graveolens*—were studied for anti-giardial activity (Table 4). The aims of these studies involved testing for anti-*Giardia* activity, evaluating pharmacology and toxicity, determining the LD_50_ values, and validating phytoextracts for diarrhoea treatment. The animals used in the in vivo experiments varied among rats, mice, and gerbils.

The effectiveness of the phytoextracts investigated was measured based on the findings from the animals tested in each study. Phytoextract treatment efficacy was determined based on a reduction in diarrhoea, a reduction in trophozoite excretion counts, or the absence of significant toxicity with low LD_50_ values measured.

Acute toxicity assays were performed in 42.8% of the *in vivo G. duodenalis* included studies [90, 99, 103], and intestinal motility tests were used in 14.2% to evaluate safety [90], while bioassays were employed in 57.1% of studies to evaluate toxicity and biological consequences [90, 99, 103, 120]. Histological analyses (H & E staining) were employed in 28.5% of studies to evaluate tissue effects (99; 102), whereas faecal trophozoite count reduction assays (28.5%) were utilised to measure treatment efficacy [74, 120].

Among the phytoextracts studied, *Rubus liebmannii* exhibited favourable results. *Rubus liebmannii* displayed an LD_50_ value greater than 5000 mg/kg with no adverse effects or deaths observed [103]. *Alocasia indica*,* Anethum graveolens*,* Rhamnus cathartica*,* Zingiber officinale*,* Yucca baccata*, and *Cinnamon zeylanicum* also showed effectiveness (Table 4). Specifically, *Alocasia indica* reduced diarrhoea [90]. While *Anethum graveolens*,* Cinnamon zeylanicum*,* Rhamnus cathartica*, *Yucca baccata*, and *Zingiber officinale* caused a reduction in trophozoite counts [74, 102, 119, 120]. In addition, *Rubus liebmannii* and *Rhamnus cathartica* did not display significant toxicity and had a low LD_50_ [102, 103]. *Alocasia indica* also exhibited an LD_50_ greater than 2000 mg/kg [90]. On the other hand, *Phaseolus vulgaris* showed no effectiveness when tested on rats [99].

The aqueous and ethanol extracts of *Alocasia indica* had IC_50_ values of 4.12 and 4.65 µg/mL, respectively [90]. These values displayed effectiveness at smaller dosages, but the toxicity effects of these two plants on the rats were not studied. *Rhamnus cathartica* had an EC_50_ value of 12 µg/mL, and no toxic effects were observed in the mice [102]. *Phaseolus vulgaris* displayed a higher toxicity with an LD_50_ value of 250 mg/kg [99]. Finally, *Anethum graveolens* was the only phytoextract tested on humans instead of animals, and it was reported to be effective in pediatric patients [119]. Alternatively, *Yucca baccata* exhibited marked toxicity, resulting in weight loss and three fatalities in gerbils, indicating that it is not a favourable therapeutic option [120]. Some phytoextracts, such as *Alocasia indica* [90], *Cinnamon zeylanicum* [74], *and Zingiber officinale* [74], have been reported to induce side effects in various animals. The side effects of the phytoextracts included mucosal irritation, reduction in goblet cells, mild discomfort, and decreased motility in the gastrointestinal tract [74, 90]. *Rubus liebmannii* and *Rhamnus cathartica* were the only two phytoextracts that were effective with no side effects [102, 103].

## Discussion

*Cryptosporidium* spp. exhibit several biological and metabolic characteristics that complicate treatment while simultaneously revealing potential therapeutic vulnerabilities. The parasite has a highly reduced metabolism, lacking de novo synthesis of many essential biomolecules and relying extensively on host-derived nutrients, alongside a remnant mitochondrial organelle (mitosome) with limited metabolic function [121, 122]. These features restrict conventional drug targets and contribute to the limited efficacy of existing therapies, particularly in immunocompromised individuals, where nitazoxanide shows inconsistent outcomes [123, 31]. Epidemiologically, cryptosporidiosis remains a leading cause of moderate-to-severe diarrhoea in young children and immunosuppressed populations worldwide, especially in low- and middle-income countries [124, 125]. Several phytoextracts demonstrating activity against *Cryptosporidium* spp. are characterized by a high content of phenolic compounds. Specifically, *Allium sativum*, *Castanea sativa*, *Schinopsis* spp., *Zingiber officinale*, and *Punica granatum* are known to contain tannins [73, 112, 117]. Nekka-Rich, a formulation derived from wood vinegar and oak bark, is particularly rich in phenols and tannins [126, 127], while *Diospyros sumatrana* contains quinones, a subclass of phenolic compounds [128]. In addition, *Olea europaea*, *Ficus carica*, *Podocarpus* sp., and *Thymus vulgaris* have been identified as sources of phenols and flavonoids [114, 116, 118], and *Cocos nucifera* L. contains a combination of flavonoids, phenols, and tannins [129]. These observations suggest that the anti-*Cryptosporidium* activity of these phytoextracts is likely mediated, at least in part, by phenolic compounds, tannins, and flavonoids, which have been widely reported to exhibit anti-parasitic properties [82, 112, 130].

*G. duodenalis* displays distinct metabolic and cellular features that influence both disease pathogenesis and therapeutic susceptibility. The parasite lacks classical mitochondria and depends on anaerobic metabolism within mitosome-containing cells, with redox-regulated pathways and cytoskeletal organization playing key roles in attachment and survival in the intestinal lumen [131, 132]. Current treatment relies mainly on nitroimidazoles and benzimidazoles; however, increasing drug resistance, adverse effects, and treatment failures have been reported in both endemic and non-endemic settings [12, 133]. Giardiasis remains globally prevalent, particularly among children and socioeconomically vulnerable populations [134]. The purified compound epicatechin isolated from *Rubus liebmannii* demonstrated notable anti-*Giardia* activity in vitro [103]. In contrast, only the crude ethanol extract of *R. liebmannii* was evaluated in vivo, suggesting that epicatechin may substantially contribute to the therapeutic effects observed in animal models. Epicatechin is a polyphenolic compound belonging to the flavonoid class [103, 130]. Similarly, other phytoextracts showing anti-*Giardia* activity-derived from *Alocasia indica*, *Anethum graveolens*, *Rhamnus cathartica*, *Zingiber officinale*, *Yucca baccata*, and *Cinnamomum zeylanicum*-are also known to contain flavonoids [74, 90, 119, 120, 135]. Within this biological framework, phytochemicals such as flavonoids and other polyphenols may exert anti-*Giardia* effects by interfering with redox homeostasis, membrane function, and cytoskeletal dynamics-mechanisms consistent with the parasite’s metabolic vulnerabilities [130, 136]. The frequent identification of flavonoid-rich phytoextracts with measurable giardicidal activity therefore supports the relevance of these parasite-specific targets.

The apparent efficacy of phytoextracts is strongly influenced by the experimental assays employed, highlighting the need for standardized evaluation frameworks to enable meaningful therapeutic assessment of plant-derived candidates. In addition, variations in routes of administration, extraction procedures, and solvent systems may substantially affect observed activity against both parasites. Across the reviewed studies, fractionation of crude extracts frequently resulted in enhanced efficacy, underscoring the importance of isolating bioactive constituents.

Previous studies on *Cryptosporidium* spp. and *Giardia duodenalis* have largely focused on narrative accounts of selected plant extracts, highlighting traditional uses and isolated in vitro or in vivo effects with limited systematic assessment of potency or safety [137, 138]. In contrast, this review provided a structured synthesis of available evidence, revealing considerable heterogeneity in cryptosporicidal activity. Only a limited number of extracts, including *Curcuma longa*, *Piper nigrum*, *Embelia ribes*, and *Nigella sativa*, demonstrated high in vitro potency, whereas many frequently cited plants exhibited minimal activity under controlled conditions [67]. Similarly, for *G. duodenalis*, efficacy varied widely, and strong in vitro activity did not consistently predict in vivo suitability. A small subset of extracts, particularly *Rubus liebmannii* and *Rhamnus cathartica*, showed both significant antiparasitic activity and favorable safety profiles, highlighting the necessity of integrating potency and toxicity assessments to ensure translational relevance [102, 103].

The authors acknowledge several limitations in the current review: (i) substantial heterogeneity existed across study designs, experimental models, and outcome measures, hindering direct comparisons and quantitative synthesis; (ii) the evidence base is largely comprised of in vitro studies, with relatively few in vivo investigations and almost no human clinical data, limiting translational applicability; (iii) most *Cryptosporidium* research focused solely on *C. parvum*, restricting species-level generalizability; (iv) inconsistencies in reporting potency metrics (IC_50_/EC_50_), variations in plant parts, extraction methods, and solvents, along with incomplete phytochemical characterization, further impede reproducibility and mechanistic understanding; and (v) toxicity evaluations were often short-term and non-standardized, despite some studies reporting adverse effects. Future research, therefore, should focus on standardizing experimental designs and potency metrics to improve comparability and reproducibility. Expanding in vivo studies and clinical trials, including a broader range of *Cryptosporidium* species, will enhance translational relevance. Comprehensive phytochemical characterization, mechanistic investigations, and long-term toxicity assessments are needed to clarify efficacy and safety. Additionally, developing advanced formulations and exploring combination therapies could improve bioavailability and therapeutic potential of plant-derived antiparasitic agents.

## Conclusions

This review shows that many plant extracts have been studied for their effects on *Cryptosporidium* and *Giardia* in the last sixty years. There is more research about *Giardia* than about *Cryptosporidium*. Some plant extracts worked in lab tests, and a few also helped animals by reducing parasite numbers or their symptoms. Extracts with high levels of certain plant chemicals, such as phenols and flavonoids, showed these effects most often.

However, most of this research is still at an early stage. Results mostly come from different types of lab tests or small-animal studies, with little agreement on how the extracts were prepared, how much was given, what was measured, or how safety was assessed. For *Cryptosporidium*, research focuses almost only on one species, *C. parvum*, and for *Giardia*, most studies target the active form of the parasite, making it hard to know if results apply broadly. Some plants, such as garlic, ginger, turmeric, and *Rubus liebmannii*, appear in several studies and seem to work better, but there isn’t enough data yet to say how well these plant extracts work, how safe they are, or whether they could be used in people.

In summary, these results suggest that plant extracts could be a useful source of treatments for cryptosporidiosis and giardiasis, but they are currently only early ideas, not proven treatments. Careful research, with clear methods, thorough chemical analysis, well-controlled animal studies, and carefully planned human trials, is needed before any plant extract can be used as an evidence-based treatment for these diseases.

## Data Availability

All relevant data are included in the paper or its Supplementary Information.
